# ChatGPT-4 Can Help Hand Surgeons Communicate Better With Patients

**DOI:** 10.1016/j.jhsg.2024.03.008

**Published:** 2024-04-06

**Authors:** Robert Browne, Khadija Gull, Ciaran Martin Hurley, Ryan M. Sugrue, John Barry O’Sullivan

**Affiliations:** ∗ Royal College of Surgeons in Ireland, Dublin, Ireland; † Department of Reconstructive and Plastic Surgery, Connolly Hospital Blanchardstown, Dublin, Ireland

**Keywords:** Artificial intelligence, ChatGPT, Communication, Health equity, Readability

## Abstract

The American Society for Surgery of the Hand and British Society for Surgery of the Hand produce patient-focused information above the sixth-grade readability recommended by the American Medical Association. To promote health equity, patient-focused content should be aimed at an appropriate level of health literacy. Artificial intelligence–driven large language models may be able to assist hand surgery societies in improving the readability of the information provided to patients. The readability was calculated for all the articles written in English on the American Society for Surgery of the Hand and British Society for Surgery of the Hand websites, in terms of seven of the commonest readability formulas. Chat Generative Pre-Trained Transformer version 4 (ChatGPT-4) was then asked to rewrite each article at a sixth-grade readability level. The readability for each response was calculated and compared with the unedited articles. Chat Generative Pre-Trained Transformer version 4 was able to improve the readability across all chosen readability formulas and was successful in achieving a mean sixth-grade readability level in terms of the Flesch Kincaid Grade Level and Simple Measure of Gobbledygook calculations. It increased the mean Flesch Reading Ease score, with higher scores representing more readable material. This study demonstrated that ChatGPT-4 can be used to improve the readability of patient-focused material in hand surgery. However, ChatGPT-4 is interested primarily in sounding natural, and not in seeking truth, and hence, each response must be evaluated by the surgeon to ensure that information accuracy is not being sacrificed for the sake of readability by this powerful tool.

The American Society for Surgery of the Hand (ASSH) and British Society for Surgery of the Hand (BSSH) have both developed patient-focused information about common hand conditions and their management on their respective websites.^1^^,^^2^ To be effective, this content should be understandable to patients of all levels of health literacy. The majority of Americans have a reading level of around sixth to eighth grade.^3^ Because of this, the American Medical Association recommends that all patient-focused educational materials be written at or below a sixth-grade readability level.^3^ This helps ensure that patients are properly informed about their condition and treatment options and promotes health equity. The issue of patient-directed content in hand surgery being aimed at a readability level above that recommended by the American Medical Association has been highlighted before,^4^ with little improvement seen over the past decade.^5^

Chat Generative Pre-Trained Transformer version 4 (ChatGPT-4) is an artificial intelligence (AI)–driven large language model that has shown great promise in many disparate fields, including health care, since its release by OpenAI (OpenAI Inc). Although it is rapidly progressing, ChatGPT has yet to surpass residents’ ability to pass hand surgery examinations^6^ and produces relatively less readable^7^ and less reliable^8^ patient-focused content than is available on professional hand society websites. However, ChatGPT has shown promise in improving the readability of patient education materials in many surgical specialties, including orthopedic surgery, when prompted to do so.^9^ The aim of this study was to evaluate the ability of ChatGPT-4 to simplify patient-focused hand surgery information provided on the ASSH and BSSH websites to a sixth-grade readability level.

## Methods

All articles written in English from the websites of the ASSH^1^ and BSSH^2^ were downloaded and converted to plain with all figures and figure legends removed.

### Readability calculation

A freely available online readability calculator, available at http://www.readabilityformulas.com, was used to calculate the readability of the included articles, before and after ChatGPT-4 alteration. This calculator determines the readability of a given piece of text based on seven commonly used readability formulas: the Automated Readability Index, Gunning Fog Score, Flesch Kincaid Grade Level (FKGL), Flesch Reading Ease (FRE), Coleman-Liau Index, Simple Measure of Gobbledygook (SMOG), and Linsear Write Formula. All of these formulas express readability in terms of grade level, with the exception of the FRE, which reports an index score. Higher index scores represent more readable material, with a score of 100 representing the most readable and 0 representing the least readable material.

### ChatGPT-4 alteration

The plain text articles were copied into ChatGPT-4 (https://chat.openai.com/), preceded by the prompt “Rewrite the following text at a sixth-grade readability level.” The first response to each prompt was copied, and readability was calculated as described above.

### Statistical analysis

Data were collected and stored in Microsoft Excel (Microsoft Corp) using a predefined proforma to include all calculated readability grade levels, index scores, and word counts, both pre- and post-ChatGPT-4 alteration. Descriptive statistics were generated for grade level measures of central tendency. Paired Student *t* tests were used to compare the pre- and post-ChatGPT-4 grade level and index scores. Significance level was set at 5%, and a two-tailed test of significance was used.

## Results

One hundred seventeen articles were included in the final analysis, 95 published by the ASSH and 22 published by the BSSH. Table 1 displays the unedited and chatGPT-4–edited mean grade levels based on each calculated readability formula. The mean decrease after ChatGPT-4 alteration was two grade levels. A readability level of grade nine was the mode in the unedited articles, with a mode of six in the ChatGPT-4–edited articles. The mean (± SD) FRE in the pre- and post-ChatGPT-4–edited text samples was 61.6 (±7.5) and 76.9 (± 8.2), respectively (*P* < .001). The mean word counts were 728.3 unedited and 303.6 for the ChatGPT-4–edited articles (*P* < .001). Table 2 shows a typical example of an article and the corresponding ChatGPT-4 version, with the mean readability of the six formulas that report grade level displayed.

**Table 1:** Mean Grade Level Per Readability Formula

Formula	Unedited (Mean∗ ± SD)	ChatGPT-4 Edited (Mean∗ ± SD)	*P* Value
**ARI**	8.7 ± 1.5	7.1 ± 1.7	< .001
**GFI**	11.0 ± 1.6	8.6 ± 1.5	< .001
**FKGL**	8.3 ± 1.4	6.0 ± 1.4	< .001
**CLI**	10.0 ± 1.3	8.0 ± 1.6	< .001
**SMOG**	8.1 ± 1.1	6.1 ± 1.2	< .001
**LWF**	8.5 ± 2.1	7.1 ± 1.7	< .001

ARI, Automated Readability Index; GFS, Gunning Fog Score; FKGL, Flesch Kincaid Grade Level; CLI, Coleman-Liau Index; SMOG, Simple Measure of Gobbledygook; LWF, Linsear Write Formula.

∗ Expressed in terms of grade level.

**Table 2:** Sample of Unedited and ChatGPT-4-Edited Text From the ASSH Website

Unedited—9th Grade Overall Mean Readability	ChatGPT-4 Edited—5th Grade Overall Mean Readability
Paraffin wax bath^1^ A paraffin wax unit is a machine that heats and holds paraffin wax, a type of wax used for candles. The wax is intended to completely cover the hand (or other body parts such as the feet). Its warm temperature is meant to provide relief from arthritis pain, sore joints or sore muscles. A paraffin wax bath is recommended for various medical and surgical hand problems and may be recommended by your hand specialist. This unit should not be used if you have open wounds/cuts, problems with sensation, or if you only want to warm your hands. A paraffin wax unit may be rented or purchased. The units are low-cost and can be purchased from a local retailer (drugstore, department store or discount store) or online. How to Give Yourself a Paraffin Wax Bath First, choose a place in your home where the unit can be set up according to the instructions. Pay particular attention to the safety features to prevent accidents. These units usually can remain set up and plugged in at all times, allowing the paraffin to be melted and available for use throughout the day. When using your unit, follow these instructions: 1. Wash your hands with soap and water and dry them. 2. Rub lotion onto your hands: Hand lotion allows the wax to be removed easily after treatment. 3. Dip your hand into the wax: Your fingertips should go in first. Keep your fingers separated and submerse your hand all the way past the wrist if desired. 4. Remove your hand after it has been coated with wax. 5. Repeat steps 3 and 4: Dip your hand 6-8 times, waiting a few seconds between each dip. This allows layers of wax to form over your hand. 6. Immediately cover your hand with a plastic bag and wrap with a hand towel: Wait 10-15 minutes. This will create moist, deep heat for your hand. 7. Remove the towel, plastic bag and cooled wax after 10-15 minutes. 8. Return the wax from your hand to the paraffin unit for reuse. 9. Proceed with exercises that have been recommended by your hand surgeon and/or hand therapist. When the wax level of your unit is low, you can purchase additional blocks of paraffin, usually from the store of your original purchase or a local beauty supply shop.	Paraffin wax bath A paraffin wax machine is like a special pot that heats up wax, which is the same kind used in candles. You can dip your hands (or feet) into this warm wax to help with arthritis pain or sore muscles. It’s a treatment that doctors might suggest for hand problems. But don’t use it if you have cuts, can’t feel well in your hands, or just want to warm them up. Here’s how you do a paraffin wax treatment: - Set up the machine safely at home. - Wash and dry your hands, then put lotion on them. - Dip your hand in the wax, fingers first. Do this 6-8 times, letting the wax build up. - Cover your hand with a plastic bag and then a towel, and wait 10-15minutes. - Take off the towel, bag, and wax. - Put the used wax back in the machine. - Do any hand exercises you doctor gave you. You can buy the machine at stores or online, and get more wax from the store or a beauty shop when you need it.

## Discussion

This article highlights the potential of AI to promote equity in the care of hand surgery patients by adjusting patient-focused materials to a readability level appropriate to their level of health literacy. Chat Generative Pre-Trained Transformer version 4 was successful in terms of FKGL and SMOG in simplifying the included texts to a mean sixth-grade readability level but unsuccessful for the four other included readability tests that report grade level. There was a statistically significant mean decrease of two grade levels per article, however.

Kirchner et al^9^ evaluated the ability of an earlier, free version of ChatGPT (9 January 2023, version 3.5) to simplify online patient educational materials about common orthopedic conditions and procedures. They prompted ChatGPT-3.5 to “translate to fifth-grade reading level(sic)” and found that the median FKGL for included articles about lumbar disc herniation, scoliosis, and spinal stenosis decreased from 9.5, 12.6, and 10.9 to 5.0, 5.6, and 6.9, respectively.^9^ They found similar results for total hip and knee arthroplasty.^9^ Kirchner et al also commented that ChatGPT3.5 achieved improved readability without any factual inaccuracies while retaining sufficient information to allow patients to make treatment decisions. This assertion was reached by each author independently reviewing the AI-converted text and coming to a conclusion, without reference to a particular quality assessment instrument.

Rouhi et al^10^ used a similar method of “independent review by multiple investigators” to identify factual errors or inaccuracies in ChatGPT-3.5 adjusted patient education materials about aortic stenosis and could find none. They similarly found a baseline higher than the recommended readability level in online patient-focused materials in aortic stenosis and a trend toward improved readability after ChatGPT-3.5 alteration. They used the FRE, FKGL, GFI, and SMOG readability scores and found that the FRE and FKGL reached their sixth-grade readability threshold.^10^

This study, as with those discussed above, is limited by the lack of an objective method of assessing the quality of material produced by ChatGPT-4. Some authors advocate tools such as DISCERN (http://www.discern.org.uk) to assess information quality in this context. This instrument is limited in the authors’ opinion as it was not validated for use in AI-generated text. Two of the 16 questions in DISCERN ask whether the sources are cited and if the date of production is clear. These are clearly irrelevant in the case of a large language model like ChatGPT-4, which has been trained on a vast quantity of source material, is constantly progressing in response to inputs, and produces answers on demand. Future work could focus on producing an instrument validated in this context.

Hand surgery is an innovative specialty, and hand surgeons are often keen to adopt new technologies to improve patient care and outcomes. However, ChatGPT-4 is optimized to provide natural-sounding responses to prompts and is not necessarily truth-seeking. Indeed, a footnote on the ChatGPT website reads: “ChatGPT can make mistakes. Consider checking important information.” As AI technology continues to rapidly evolve, it is likely that the barrier to entry will be lowered for individual organizations like the ASSH and BSSH to create personalized chatbots that can interact with patients, providing accurate information at a readability level appropriate to their level of health literacy. Until then, this study has demonstrated that ChatGPT-4 can be a powerful tool in improving the readability of patient-focused educational materials, but it remains incumbent upon individual surgeons who use this technology to ensure the accuracy of the information provided.

## Declaration of Generative AI and AI-assisted Technologies in the Writing Process

During the preparation of this work, the authors used ChatGPT-4 in order to adjust the articles of text as explicitly described in the manuscript above but was not used in drafting or adjusting the remaining manuscript. After using this tool/service, the authors reviewed the content and analyzed it as described in the manuscript and take full responsibility for the content of the publication.

## Conflicts of Interest

No benefits in any form have been received or will be received related directly to this article.

## References

[1] ASSH The American Society for Surgery of The Hand—Handcare. https://www.assh.org/handcare.

[2] BSSH The British Society for Surgery of the Hand—Patients. https://www.bssh.ac.uk/patients/.

[3] Weiss B.D. (2007).

[4] Mertz K., Burn M.B., Eppler S.L., Kamal R.N. (2019). The reading level of surgical consent forms in hand surgery. J Hand Surg Global Online.

[5] Zhang D., Earp B.E., Kilgallen E.E., Blazar P. (2022). Readability of online hand surgery patient educational materials: evaluating the trend since 2008. J Hand Surg.

[6] Han Y., Choudhry H.S., Simon M.E., Katt B.M. (2024). ChatGPT’s performance on the hand surgery self-assessment exam: a critical analysis. J Hand Surg Global Online.

[7] Crook B.S., Park C.N., Hurley E.T., Richard M.J., Pidgeon T.S. (2023). Evaluation of online artificial intelligence-generated information on common hand procedures. J Hand Surg.

[8] Christy M., Morris M.T., Goldfarb C.A., Dy C.J. (2024). Appropriateness and reliability of an online artificial intelligence platform responses to common questions regarding distal radius fractures. J Hand Surg.

[9] Kirchner G.J., Kim R.Y., Weddle J.B., Bible J.E. (2023). Can artificial intelligence improve the readability of patient education materials?. Clin Orthop Relat Res.

[10] Rouhi A.D., Ghanem Y.K., Yolchieva L. (2024). Can artificial intelligence improve the readability of patient education materials on aortic stenosis? A pilot study. Cardiol Ther.

